# Virtual Reality, Augmented Reality, Gamification, and Telerehabilitation: Psychological Impact on Orthopedic Patients’ Rehabilitation

**DOI:** 10.3390/jcm9082567

**Published:** 2020-08-07

**Authors:** Alessandra Berton, Umile Giuseppe Longo, Vincenzo Candela, Sara Fioravanti, Lucia Giannone, Valeria Arcangeli, Viviana Alciati, Claudia Berton, Gabriella Facchinetti, Anna Marchetti, Emiliano Schena, Maria Grazia De Marinis, Vincenzo Denaro

**Affiliations:** 1 Department of Orthopedic and Trauma Surgery, Campus Bio-Medico University, Via Alvaro del Portillo, 200, Trigoria, 00128 Rome, Italy; a.berton@unicampus.it (A.B.); v.candela@unicampus.it (V.C.); denaro@unicampus.it (V.D.); 2 Research Unit Nursing Science, Campus Bio-Medico di Roma University, 00128 Rome, Italy; dsoke.cbm@gmail.com (S.F.); mazzella.cbm@gmail.com (L.G.); fusilli.cbm@gmail.com (V.A.); fumo.cbm@gmail.com (V.A.); facchinetti.cbm@gmail.com (G.F.); marchetti.cbm@gmail.com (A.M.); demarinis.cbm@gmail.com (M.G.D.M.); 3 School of Physiotherapy, Tor Vergata University of Rome, Via Orazio Raimondo 18, 00173 Rome, Italy; claudia.certon87@gmail.com; 4 Laboratory of Measurement and Biomedical Instrumentation, Campus Bio-Medico University, 00128 Rome, Italy; e.schena@unicampus.it

**Keywords:** rehabilitation, physiotherapy, remote rehabilitation, virtual rehabilitation, virtual reality, augmented reality, gamification, telerehabilitation, orthopedic, hip, knee, shoulder, elbow, wrist, hand, humerus, femur, spine, ankle, foot

## Abstract

Background: Remote virtual rehabilitation aroused growing interest in the last decades, and its role has gained importance following the recent spread of COVID19 pandemic. The advantages of virtual reality (VR), augmented reality (AR), gamification, and telerehabilitation have been demonstrated in several medical fields. In this review, we searched the literature for studies using these technologies for orthopedic rehabilitation and analyzed studies’ quality, type and field of rehabilitation, patients’ characteristics, and outcomes to describe the state of the art of VR, AR, gamification, and telerehabilitation for orthopedic rehabilitation. Methods: A comprehensive search on PubMed, Medline, Cochrane, CINAHL, and Embase databases was conducted. This review was performed according to PRISMA guidelines. Studies published between 2015 and 2020 about remote virtual rehabilitations for orthopedic patients were selected. The Methodological Index for Non-Randomized Studies (MINORS) and Cochrane Risk-of-Bias assessment tool were used for quality assessment. Results: 24 studies (9 randomized controlled trials (RCTs) and 15 non-randomized studies) and 2472 patients were included. Studies mainly concern telerehabilitation (56%), and to a lesser extent VR (28%), AR (28%), and gamification (16%). Remote virtual technologies were used following knee and hip arthroplasty. The majority of included patients were between 40 and 60 years old and had a university degree. Remote virtual rehabilitation was not inferior to face-to-face therapy, and physical improvements were demonstrated by increased clinical scores. Orthopedic virtual remote rehabilitation decreased costs related to transports, hospitalizations, and readmissions. Conclusion: The heterogeneity of included studies prevented a meta-analysis of their results. Age and social context influence adaptability to technology, and this can modify compliance to treatment and outcomes. A good relationship between patient and physiotherapist is essential for treatment compliance and new technologies are useful to maintain clinical interactions remotely. Remote virtual technologies allow the delivery of high-quality care at reduced costs. This is a necessity given the growing demand for orthopedic rehabilitation and increasing costs related to it. Future studies need to develop specific and objective methods to evaluate the clinical quality of new technologies and definitively demonstrate advantages of VR, AR, gamification, and telerehabilitation compared to face-to face orthopedic rehabilitation.

## 1. Introduction

Orthopedic rehabilitation is of paramount importance after a trauma or surgery to recover impaired function [1]. A successful therapy requires the appropriate combination and progression of exercises to improve joint mobilization and muscle strengthening to recover physical function [1]. The rehabilitation program begins immediately after surgery in the hospital setting and then proceeds in a private/home setting [2]. Current modalities of rehabilitation include both supervised and unsupervised exercises, but advances in technology are opening new horizons in this field. Virtual reality (VR), augmented reality (AR), gamification, and telerehabilitation are appealing for orthopedic patients’ rehabilitation.

VR and AR aim to deceive the brain of patients by making them believe that they are in other places than the real one [3]. In VR, the patient interacts with a virtual environment and simulates activity of real life. The risk of this technology is the impossibility to recognize real dangers that can cause injuries. In AR, virtual reality and real reality overlap and the patient is aware of potential dangers [4].

The concept of gamification is based on the application of “game design elements in a non-game context” to motivate participation [5]. Beneficial effects have been reported in several fields of disabilities (e.g., idiopathic scoliosis and stroke rehabilitation) [3].

Telerehabilitation is a branch of telemedicine that allows patients to communicate with their health care provider remotely during rehabilitation session [6].

These technologies reduce patient hospitalization times and costs and increase the number of patients who can be treated at the same time [7]. Another positive aspect of these rehabilitative modes is direct and continuous interaction between the patient and the health care provider, which increases compliance to treatment [6]. Studies demonstrated that remote virtual rehabilitation enhances patient’s motivation improving adherence to therapy [8].

Despite these benefits, there are still many aspects of remote technologies for orthopedic rehabilitation that need to be analyzed and definitive conclusions about their advantages compared to face-to-face rehabilitation still need to be achieved [9].

In this review, we searched the literature for studies using remote virtual technologies for orthopedic rehabilitation and analyzed studies’ quality, type and field of rehabilitation, patients’ characteristics, and outcomes to describe the state of the art of VR, AR, gamification, and Telerehabilitation for orthopedic rehabilitation.

## 2. Materials and Methods

Literature search was performed according to PRISMA guidelines [10]. Preliminary searches of primary databases could not find any existing or ongoing systematic reviews about remote virtual rehabilitation for orthopedic patients.

### 2.1. Eligibility Criteria and Search Strategy

Keywords and combinations of keywords were used to search electronic databases and were organized according to the Population, Intervention, Comparison, Outcome (PICO) model as follows.

Study: original studies with different study designs (randomized controlled trials, prospective studies, retrospective analysis, pilot randomized controlled trial, narrative synthesis, prospective longitudinal cohort studies, feasibility study, pilot study); English language; articles published from 2015 to 2020.

Participants: orthopedic patients.

Interventions: remote virtual rehabilitations (VR, AR, gamification, and telerehabilitation). 

Outcome measures: the primary outcome was to evaluate quantitative outcomes of remote virtual rehabilitation for orthopedic patients. The secondary outcome was to assess qualitative outcomes, patients’ characteristics such as age and social context, and costs. Other goals were to describe fields of orthopedic surgery and disease where remote virtual technologies were used. 

A comprehensive search on PubMed, Medline, Cochrane, CINAHL, and Embase databases was conducted. Keywords were combined using the Boolean operators “AND” and “OR”. The search strategy was iterative and flexible within the limits of the search engines of the individual databases. 

The following medical subject heading (MeSH) keywords and free terms were used for the search: rehabilitation, physiotherapy, remote rehabilitation, virtual rehabilitation, virtual reality, augmented reality, gamification, telerehabilitation, orthopedic, hip, knee, shoulder, elbow, wrist, hand, humerus, femur, spine, ankle, foot.

Search strategies were checked by two reviewers (S.F. and L.G.).

Exclusion criteria included: reviews, books, and protocol study.

### 2.2. Study Selection and Data Collection

The systematic review was carried out in June 2020. Two researchers (S.F. and L.G.), independently reviewed all studies (title, abstract, and full text) that met inclusion criteria and extracted relevant data. A discussion among reviewers resolved disagreements.

### 2.3. Quality Assessment

Two reviewers (S.F. and L.G.) independently evaluated the potential risk of bias of included studies using the Methodological Index for Non-randomized Studies (MINORS) [11], and the Cochrane Risk-of-Bias Tool [12] for randomized controlled trials (RCTs).

MINORSs’ items were scored 0 if not reported, 1 when reported but inadequate, 2 when reported and adequate. The global ideal score was 16 for non-comparative studies and 24 for comparative studies.

The Cochrane Risk-of-Bias Tool assessed randomized controlled trials with the following criteria: selection, performance, detection, attrition, reporting, and other biases. All criteria were evaluated assigning 0 for low risk, 1 point for unclear, and 2 points for high risk of bias. The potential total score ranged 0–14. An overall score of 0–1 shows high quality, 2–3 moderate quality, and >3 low quality [10].

### 2.4. Data Synthesis and Analysis

Data about study design, number of patients, follow-up period, orthopedic surgery and disease, remote virtual technology used, patients’ age and social context, quantitative and qualitative outcome measures, and costs were extracted. 

Categorical variables were reported as percentage frequencies. Continuous variables were reported as mean, minimum, and maximum values.

## 3. Results

**Figure 1:**
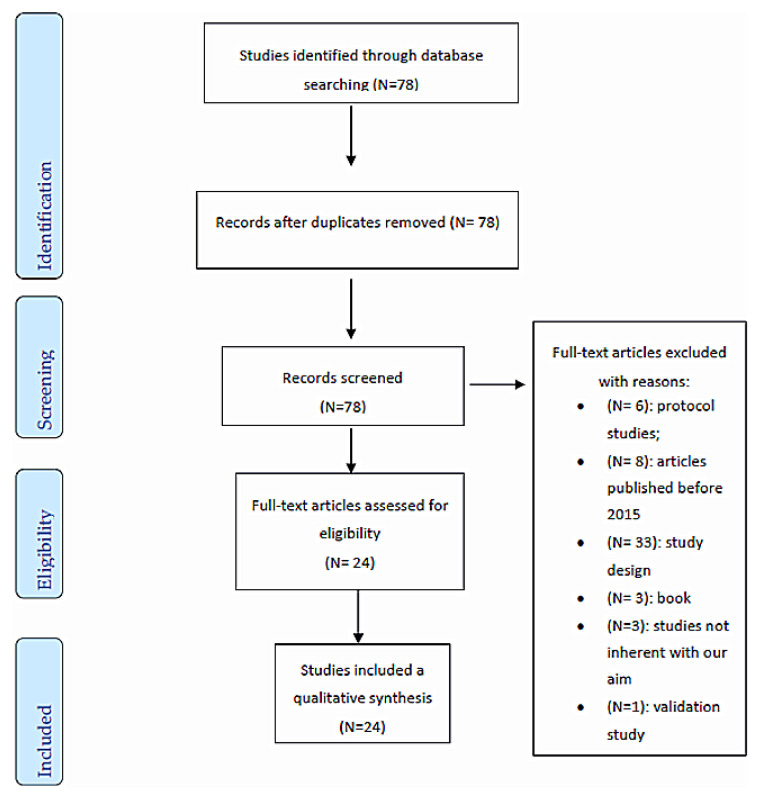
Flow chart of studies selection according to PRISMA guidelines [13].

The selection process is illustrated in Figure 1. The search strategy yielded 78 articles. After duplicate removal and title, abstract, and full-text review, 24 studies were evaluated for methodological quality and were eligible for the review.

### 3.1. Studies and Patients Characteristics

Studies included a total of 2472 of patients. Details about number of patients, study design, remote virtual rehabilitation technology, and follow-up are reported in Table 1.

The majority of the studies analyzed telerehabilitation (56%), 28% analyzed VR and AR, and 16% analyzed gamification.

The follow-up of the studies was evaluated to understand the time needed to adapt to the technology. It was the preoperative period in 8% of the studies, 3 days in 4%, 10 days in 4%, 2 weeks in 4%, 3 weeks in 4%, 1 months in 4%, 2 months in 20%, 3 months in 8%, 4 months in 20%, 6 months in 12%, 17 months in 4%, 18 months in 4%, and 21 months in 4%.

Age and social context were collected to analyze adaptability to technology in relation to these characteristics (Table 2). The majority of the studies (79%) included patients between 40 and 60 years of age. Forty-three percent had a university degree, 27% were workers, 8% were unemployed, and 0.5% were retired. Twelve percent of the study included patients between 60 and 80 years of age. Of these, 10% were retired, 2% were workers, and 0.5% were semi-retired. The third age group by frequency included patients between 25 and 30 years of age (9%), and all of them had a university degree.

Table 3 summarizes type of orthopedic rehabilitation, surgery, and disease.

Remote rehabilitation was tested mainly for total knee arthroplasty (TKA), total hip arthroplasty (THA), and unicompartmental knee arthroplasty (UKA) (73%). 

VR and AR were used for TKA (4%); chronic nonspecific low back pain (4%); and interventions of hip, knee, and ankle (1%). 

Gamification was implemented above all for rheumatoid arthritis (7%) and to a lesser extent for fracture of metacarpal (1%).

#### 3.1.1. Qualitative Results

The qualitative outcomes were used to understand how the patient qualifies the experienced with the new rehabilitation technology (Table 4).

Qualitative studies focused on costs, communication between patient and health care provider, user-friendliness, and perceived improvements.

Costs were lower, mainly thanks to the reduction in travel expenses [34].

Patients reported an increase in self-esteem themselves and an improved relationship with the physiotherapist despite the distance [32]. 

Patients complained about difficult adaptation to the new technology at the beginning, but the majority described physical improvements [16]. 

#### 3.1.2. Quantitative Outcomes

Quantitative data were used to verify physical improvements of patients (Table 5).

The Visual Analogue Scale for pain (VAS) was used in 9.4% of the studies [18,19,20,21,22,23,24,25]; Western Ontario and McMaster Universities’ Arthritis Index (WOMAC), in 8% [26,27]; Knee Injury and Osteoarthritis Outcome Score (KOOS) in 4.7% [22,27]; Time Up-and-Go Test (TUG) in 3% [27,28,29,30]; and Short-Form 36 (SF-36) in 3%.

The heterogeneity of clinical scores used in the studies did not allow the calculation of definitive quantitative outcomes.

### 3.2. Quality Assessment

Non-randomized studies (*n* = 15; 62.5%) were evaluated with MINORS. Of these studies, 10 (66.7%) had a low risk of bias, and 5 (33.3%) had a high risk of bias. 

Nine studies were RCTs. One article (11.1%) had high quality, six articles (66.6%) had moderate quality due to insufficient details about sources of bias, and two articles (22.3%) had low quality due to inadequate information about the double-blinding process and sources of bias.

## 4. Discussion

Remote virtual rehabilitation aroused growing interest in the last decades, and its role has gained importance following the recent spread of the COVID19 pandemic. 

The advantages of VR, AR, gamification, and telerehabilitation have been already demonstrated in several medical fields [35,36], but studies focused on orthopedic rehabilitation [31] are still scant. 

In this review, we searched the literature for studies using remote technologies for orthopedic rehabilitation and analyzed the studies’ quality, type and field of rehabilitation, patients’ characteristics, and outcomes to describe the state of the art of VR, AR, gamification, and telerehabilitation for orthopedic rehabilitation. 

Available studies about remote virtual rehabilitation mainly concern telerehabilitation and, to a lesser extent, VR, AR, and gamification. The reason is that gamification has been introduced only recently and only few studies have investigated it so far. The first use of VR and AR dates back to 1998, when the improved image visualization was supposed to be applied for diagnostic purposes [37]. Progress led to the use of these methods for rehabilitation and medical teaching. Moreover, telerehabilitation is more widespread because it is easier to realize compared to the others. 

Virtual technologies have been largely evaluated for remote rehabilitation following knee and hip arthroplasty. Studies usually included patients between 40 and 60 years of age, who mostly had a university degree. Age and social context influence adaptability to technology. For elderly patients, it is challenging to approach technology, while younger patients are predisposed to it [29]. Therefore, compliance to treatment and outcomes can be affected by the patient’s perception of technology. As the majority of orthopedic patients are of medium-high age, the user-friendliness of remote virtual technology should be guaranteed. More and more simple platforms have been created. They do not require complicated software or installation of multidirectional cameras. It is merely necessary that the patient has a computer or a smartphone to connect to the Internet [9]. 

The analysis of qualitative and quantitative outcomes showed increased patient self-esteem and a consolidated relationship with the physiotherapist, as well as good clinical outcomes [32]. The effectiveness of remote virtual rehabilitation in orthopedics was supported by several studies. It was not inferior to face-to-face therapy on several outcomes. Increased clinical scores demonstrated physical improvements following remote virtual rehabilitation. 

Orthopedic remote rehabilitation leads to reduced costs for the national health system. As already proven in other medical fields [34], decreased costs are related to the reduction of transports, hospitalizations, and readmissions [6,17,22,23,24,27,30,33,38]. It is assumed that for patients living more than 30 km away from the rehabilitation center, savings are around 230 dollars [34]. This system is advantageous not only for those who live far from rehabilitation centers but also for people with severe disabilities as moving is not necessary [22]. Moreover, remote virtual rehabilitation allows continuous monitoring of several patients at the same time, saving time and money [27,30]. The possibility to deliver high-quality care at reduced costs is necessary given the growing demand for orthopedic rehabilitation and increasing costs related to it. 

New technologies offer promise for the growing demand of orthopedic rehabilitation, but barriers and issues need to be overcome in the field. Elderly patients represent a substantial part of orthopedic patients. However, the spreading of VR, AR, gamification, and telerehabilitation is limited among patients of advanced age. This category of patients is not prone to new technologies and showed low adaptability to them. The main challenge is to improve these technologies to be accessible and suitable for this population. Their technology should be targeted, and the experience should be simplified to engage the patient. By employing machine learning, exercises can be tailored to patient’s needs and ability. Moreover, an intuitive interface can facilitate the use of virtual technologies.

Real-time monitoring of patients’ physiotherapy is another challenge. The need to analyze user activity as it is happening can be useful to prevent pitfalls during training. It can be bypassed by programmed visual-optic feedbacks for specific tasks. Future developments should focus on adequate data storage systems and real-time analysis of continuous updated information, to provide immediate feedback to patients. Those systems should also guarantee privacy protection. 

There is huge potential for remote virtual rehabilitation as shown by data reported by available studies. It is recommended that these technologies should be further improved and their fields of application should be expanded as they allow the delivery of high-quality care at reduced costs.

Future studies should establish the areas of physiotherapy that will benefit most from this technology. New digitally enabled technological solutions should be searched to underpin transformative health innovations that can have a direct benefit to patients’ rehabilitation.

The main limitation of this review is the heterogeneity of included studies that prevented a meta-analysis of their results. There were no standard procedures or protocols, and different equipment were used. Outcomes measurement methods differed between studies, and many data were qualitative rather than quantitative. 

Only nine RCTs and 15 non-randomized studies were analyzed. Nevertheless, RCTs were of high or moderate quality and the majority of the non-randomized studies had a low risk of bias. 

The literature lacks data regarding patient’s perception of new technology and adherence to therapy. Future research needs to develop specific and objective methods to evaluate the clinical quality of new technologies. 

To our knowledge, this is the first study providing an overview of remote virtual techniques for orthopedic rehabilitation. This study provides an overview of the field, highlighting the benefits of these methods. At the same time, this review underlines the need for future research to definitively demonstrate advantages of VR, AR, gamification, and telerehabilitation compared to face-to face orthopedic rehabilitation. 

## 5. Conclusions

This review evaluated literature about remote virtual technologies for orthopedic rehabilitation. Only nine RCTs and 15 non-randomized studies were available in literature, but their quality was high or moderate and the risk of bias was low. Heterogeneity of included studies prevented a meta-analysis of their results. There were no standard procedures or protocols, and different equipment and outcomes measurement methods were employed between studies. Age and social context influence adaptability to technology, and it can modify compliance to treatment and outcomes. A good relationship between patient and physiotherapist is essential for treatment compliance, and new technologies are useful to maintain clinical interactions remotely. Remote virtual technologies allow the delivery of high-quality care at reduced costs. This is a necessity given the growing demand for orthopedic rehabilitation and increasing costs related to it. Future studies need to develop specific and objective methods to evaluate the clinical quality of new technologies and definitively demonstrate advantages of VR, AR, gamification, and telerehabilitation compared to face-to face orthopedic rehabilitation. 

## Figures and Tables

**Table 1:** Characteristics of the included studies.

Author and Country	Number of Patients	Study Design	Remote Virtual Rehabilitation Technology	Follow-Up
Valenzuela 2020, UK [14]	NA	Mixed (qualitative and RCT)	Gamification	2 weeks
van der Kooij 2019, Netherlands [15]	28 for first experiment	Pseudo-randomized trial	Gamification	3 weeks
	22 for second experiment			
Then 2020, Malaysia [8]	21	RCT	Gamification	2 weeks
Allam 2015, Switzerland [5]	155	RCT	Gamification	6 months
Babic 2019, Norway [16]	6	Qualitative study	VR	0
Chan 2019, Hong Kong [4]	13	Feasibility study	VR/AR	0
Chughtai 2019, Ohio [17]	157	Non-randomized trial	VR	1 month
Gianola 2020, Italy [18]	85 but during the study lost 11 patients	RCT	VR	10 days after surgery
Matheve 2020, Belgium [19]	84	RCT	VR	21 months
Pekyavas 2017, Turkey [20]	30	RCT	VR	2 months and half
Azma, 2017, Iran [21]	54	RCT	TR	6 months
Hernandez, 2016, Mexico City [22]	20 but during the study lost 9 patients	Auto-controlled study	TR	6 months
Kuether 2019, USA [6]	654	Pilot study	TR	12 weeks
Bini 2017, USA [23]	51	RCT	TR	2 months
Correia 2018, Portugal [24]	236, final analysis 69 (37 + 32). Completed the study 30 + 29	Feasibility study	TR	8 weeks
Eichler 2019, Germany [25]	111 but during the study lost 24 patients	RCT	TR	3 months
Çubukçu 2019, Turkey [26]	40	Non-randomized control trial	TR	3 days
Doiron-Cadrin 2020, Canada [27]	34	RCT	TR	12 weeks
Tousignant 2015, Canada [28]	17	Pilot study	TR	8 weeks
Nelson 2017, Australia [29]	75	Qualitative study	TR	2 months
Chughtai 2019, USA [30]	476	Cohort study	TR	17 months
Richardson 2017, Australia [31]	18	Repeated-measures design	TR	2 months
Naeemabadi 2020, Denmark [32]	NA	Qualitative study	TR	2 weeks
Tsvyakh 2017, Ukraine [33]	74	RCT	TR	3 months

TR: telerehabilitation; VR: virtual reality; AR: augmented reality; RCT: randomized controlled trial; NA: not available.

**Table 2:** Age and social context of included patients.

Age	Social Context
25–30 (35–9%)	University degree (35–9%)
40–60 (312–79%)	Unemployed (31–8%) University degree (171–43%) Worked (108–27%) Retired (2–0.5%)
60–80 (51–12%)	Retired (41–10%) Worked (8–2%) Semi-retired (2–0.5%)

**Table 3:** Remote virtual rehabilitation technology, orthopedic surgery, and disease.

Remote Virtual Rehabilitation Technology	Orthopedic Surgery	Orthopedic Disease	Total
Gamification	Immobilization for 4 to 6 weeks	Fracture of metacarpal	21–1%
Gamification	NS	Rheumatoid arthritis	155–7%
VR	TKA	Osteoarthritis	85–4%
VR	NS	Chronic nonspecific low back pain	84–4%
VR	NS	subacromial impingement syndrome and scapular dyskinesis	30–1%
VR/AR	NS	Found in the hip, knee and ankle joint	16–1%
TR	TKA or UKA or THA	Osteoarthritis	1591–73%
TR	shoulder joint replacement	Primary osteoarthrosis or rheumatoid arthritis	10–0.5%
TR	NS	Rotator cuff tears	11–0.5%
TR	TJR	Severe hip and knee osteoarthritis	75–3%
TR	hip fracture surgery	Acute hip fracture	70–3%
TR	immobilization for 4 to 6 weeks	Proximal humerus fracture	147–7%
TR	TKR	NS	29–1%

TR: telerehabilitation; VR: virtual reality; AR: augmented reality; RCT: randomized controlled trial; TKA: total knee arthroplasty; UKA: unicompartmental knee arthroplasty; TJR: total joint replacement; TKR: total knee replacement; THA: total hip arthroplasty.

**Table 4:** Qualitative outcomes.

Author and Country	Outcome Measure	Outcome Measure Result
Bini 2017, USA [23]	PRO (questionnaire)	Patient satisfaction overall with both the traditional patient care pathway and the digital interface was high, and there was no major difference.
Nelson 2017, Australia [29]	Questionnaire Franzen and Oppenheim	Only 35% reported feeling confident using technology. The results change considerably with advancing age: Telerehabilitation is feasible from the perspective of access to, feelings toward, and preferences for technology.
Doiron-Cadrin 2020, Canada [27]	Questionnaire	All participants (100%) felt they met their rehabilitation goals, felt positive about their telerehabilitation experience, and were satisfied with their physiotherapy treatments.
Babic 2019, Norway [16]	Interview A scale from one (low) to 5 (high) to collect the feedback.	The responses were that sometimes, negative feedback concerned nausea occurring during VR, but the overall experience was positive.
Naeemabadi 2020, Denmark [32]	Interviews Questionnaires (Likert scale)	Iteration 4: The user-friendliness of the TR was high to very high. The patients reported a lower level of satisfaction in the area of communication and training with the wearable sensors. Iteration 5: The level of motivation among patients increased. A higher level of self-confidence was reported. The participants believed that physiotherapist’s feedback on the patients’ performance and questions induced a sense of security. The majority of the users claim that the system can considerably reduce the need for travel.

**Table 5:** Quantitative outcomes.

Author and Country	Outcome Measure	Outcome Measure Result
van der Kooij 2019, Netherlands [15]	EXPERIMENT 1	EXPERIMENT 1
	QMI	(1) IMI: game group scored higher than the control group: IG = −2.37, CG = 0.03
	IMI	(2) QMI: did not differ significantly between the game and control group IG = 1.82, CG = 0.068
	EXPERIMENT 2	EXPERIMENT 2
	CoM velocity;	(1) CoM velocity: effect of group F (1,37) = 0.48, *p* = 0.49, interaction of group and block F (2,74) = 1.16, *p* = 0.32, and effect of block, F (2,74) = 1.99, *p* = 0.14.
	CoM distance	(2) CoM distance: effect of block F (1.34,49.57) = 15.46 *p* < 0.001, effect of group F (1,37) = 2.32 *p* = 0.14, and interaction of group and block F (1.34,49.57) = 0.21 *p* = 0.72
	QMI	(3) QMI: z = −1.06, *p* = 0.29
Then 2020, Malaysia [8]	grip strength;	-grip strength: IG = 36.15, CG = 30.74;
	composite finger ROM;	-composite finger ROM: IG = 2.78, CG = 4.50
	PRWE;	-PRWE: IG = 3.44, CG = 8.45
	compliance (min/day)	-compliance: IG = 26.89, CG = 16.57
Allam 2015, Switzerland [5]	Exercise Behaviors Scale;	Exercise Behaviors Scale: (B = 3.39, *p* = 0.02)
	Health Care Utilization Scale;	Health care system: (B = 2.79, *p* = 0.02)
	Prescription Opioid Misuse Index;	Prescription Opioid Misuse Index: (B = 12.06, *p* = 0.03);
Chan 2019, Hong Kong [4]	CoP Ellipse area;	CoP Ellipse area: for AR 433.78 ± 229.27 (*p*), for VR 934.14 ± 745.09 (*p*);
	Stride length;	Stride length: for AR 0.98 ± 0.07 (*p*), for VR 0.98 ± 0.06 (*p*)
	Cadence	Cadence: for AR 102.41 ± 7.90 (*p*), for VR 102.73 ± 6.59 (*p*)
Chughtai 2018, Ohio [17]	KSS; SUS;	KSS pain and function scores improved, and the improvements were measured at 368% for TKA and 350% for UKA (pain) and 27% for UKA and 33% for TKA (function). Moreover, WOMAC scores improved by 57% and 66% for UKA and TKA patients, while the improvement in AM-PAC scores was at 22% and 24%.
	WOMAC;	
	AM-PAC	
Gianola 2020, Italy [18]		All the results are reported in change from before to after study
	VAS;	VAS: IG = −23.03, CG = −28.97
	WOMAC;	WOMAC: IG = −790.28, CG = −765.77
	EQ-5D;	EQ-5D: IG = 0.13, CG = 0.15
	GPE;	GPE: IG = 4.58, CG = 4.71
	FIM questionnaire	FIM: IG = 17.03; CG = 21.19
Matheve 2020, Belgium [19]	NPRS;	To evaluate pain-related fear there was a main effect for group (all *p*-values < 0.0001) and for TSK (all *p*-values < 0.02), but there was no interaction effect (all *p*-values > 0.54). To evaluate pain catastrophizing, a main effect for group (all *p*-values < 0.0001) and PCS (all *p*-values < 0.02) was present, but there was no interaction effect (all *p*-values > 0.5)
	RMDQ;	
	PCS;	
	TSK	
Pekyavas 2017, Turkey [20]	SPADI;	SPADI: IG = 8.13, CG = 11.41;
	NEER;	NEER: IG = 0.00, CG = 0.08;
	HAWKINS;	HAWKINS: IG = 0.00, CG = 0.00;
	SRT;	SRT: IG = 2.22, CG = 0.08;
	SAT;	SAT: IG = 0.00, CG = 0.83;
	LSST	LSST: IG = 0.42, CG = 1.58
Azma 2017, Iran [21]	VAS;	VAS (62.5 ± 9.1);
	WOMAC;	WOMAC (72.5 ± 23.2)
	KOOS	KOOS (79.4 ± 28.3);
Macías-Hernández 2016, Mexico City [22]	VAS;	VAS pain (after 6 months 16 (0–30));
	CM	CM (after 6 months 85 (70–100))
Kuether 2019, USA [6]	KOORS;	KOOS 4.6.
	HOOS	HOOS 4.4;
Bini 2017, USA [23]	VAS;	VAS (−3.724);
	VR-12;	VR-12 PCS (15.310); VR-12 MCS (3.611).
	KOOS-PS	KOOS (−17.415);
Çubukçu 2019, Turkey [26]	Degree (Clinical goniometer and digital goniometer)	Clinical goniometer vs. Kinect V2: 0.33° (abduction), −2.83° (flexion), −0.50° (external rotation), −6.67° (internal rotation) and −0.10° (extension). Digital goniometer vs. Kinect V2: 1.10° (abduction), −1.63° (flexion), −0.38° (external rotation), −5.35° (internal rotation) and 0.03° (extension).
Eichler 2019, Germany [25]	WOMAC;	WOMAC (IG −14.9, CG −10.9);
	SF-36;	SF-36 PCS (IG 10.7, CG 11.1); SF-36 MCS (IG −2.5, CG 0.1)
Doiron-Cadrin 2020, Canada [27]	SPW; ST; WOMAC; SF-36; LEFS; TUG; GRC	LEFS (pre-TR 2.9 ± 13.9, in-person pre-rehab −2.6 ± 6.7, control −1.3 ± 11.1); WOMAC pain (pre-TR −0.3 ± 4.8, in-person pre-rehab −0.8 ± 2.8, control 0.5 ± 2.8); SF-36 PCS (pre-TR −0.5 ± 7.0, in-person pre-rehab 0.2 ± 7.0, control −0.4 ± 5.2); SF-36 MCS (pre-TR 1.0 ± 10.0, in-person pre-rehab 0.5 ± 8.0, control −1.0 ± 8.5); TUG (pre-TR −0.8 ± 1.7, in-person pre-rehab −0.2 ± 1.7, control 0.3 ± 1.5); SPW (pre-TR −5.0 ± 5.3, in-person pre-rehab −4.2 ± 5.4, control 0.9 ± 12.3); ST (pre-TR −2.1 ± 2.7, in-person pre-rehab −2.0 ± 5.7, control −2.6 ± 8.0);
Richardson 2017, Australia [31]	VAS;	VAS (« a high overall reporting of satisfaction ») Validity: 2 different/18 4 similar/18 Intra-rater reliability: 89% Inter-rater reliability: 67%
Tousignant 2015, Canada [28]	SF-MPQ;	SF-MPQ (10.6 ± 12.4);
	VAS;	VAS (26.3 ± 21.8);
	F-DASH;	F-DASH (42.1 ± 11.4).
	questionnaire	Questionnaire (global score 82 ± 7%)
Tsvyakh 2017, Ukraine [33]	LEFS	IG 44,62; CG 36.43
Chughtai 2019, USA [30]	LOS	LOS 2.0 rehab; 2.7 GC
Correia 2018, Portugal [24]	TUG; KOOS	TUG (IG: −9.5, CG: −4.6)

IG: intervention group; CG: control group; TR: telerehabilitation; VR: virtual reality; AR: augmented reality; RCT: randomized controlled trial; TKA: total knee arthroplasty; UKA: unicompartmental knee arthroplasty; TJR: total joint replacement; TKR: total knee replacement; QMI: Quality of Marriage Index; IMI: Intrinsic Motivation Inventory; PRWE: Patient-Rated Wrist Evaluation; ROM: range of motion; KSS: Karolinska Sleepiness Scale; SUS: System Usability Scale; WOMAC: Western Ontario and McMaster Universities’ Arthritis Index; AM-PAC: Activity Measure for Post-Acute Care; EQ-5D: health-related quality of life; GPE: global perceived effect; FIM: functional independence measure; NPRS: Numeric Pain Rating Scale; RMDQ: Roland–Morris Disability Questionnaire; PCS: pain catastrophizing; TSK: Tampa Scale of Kinesiophobia; SPADI: Shoulder Pain and Disability Index; NEER: Neer test; HAWKINS: Hawkins Scale; LSST: lateral scapular slide test; VAS: Visual Analogue Scale; KOOS: Knee Injury and Osteoarthritis Outcome Score; CM: Constant–Murley score; KOORS: Knee injury and Osteoarthritis Outcome Score, Junior; HOOS: Hip disability and Osteoarthritis Outcome Score; SF-36: Short-Form 36; LEFS: Lower Extremity Functional Scale; TUG: Timed Up-and-Go Test; SF-MPQ: Short-Form McGill Pain Questionnaire; F-DASH: Disability of the Arm, Shoulder, and Hand; CoM: center of mass; CoP: center of pressure; SRT: Scapular Retraction Test, SAT: Scapular Assistance Test; VR-12: health survey; ST: Stair Test; LOS: length of stay; SPW: self-paced; MCS: mental component scores.
